# CT features and quantitative analysis of subsolid nodule lung adenocarcinoma for pathological classification prediction

**DOI:** 10.1186/s12885-020-6556-6

**Published:** 2020-01-28

**Authors:** Xiaohu Li, Wei Zhang, Yongqiang Yu, Guihong Zhang, Lifen Zhou, Zongshan Wu, Bin Liu

**Affiliations:** 1 Department of Radiology, the First Affiliated Hosptial of Anhui Medical University, No.218 Jixi Road, Hefei, 230022 Anhui China; 2 Department of Radiology, The Lu’an affiliated hospital, Anhui Medical University, No.21wanxi Road, Luan, Anhui China; 3 Department of Pathology, the First Affiliated Hosptial of Anhui Medical University, No.218 Jixi Road, Hefei, Anhui China

**Keywords:** Lung adenocarcinoma, Ground glass, Computed tomography, Pathology, Image features;morphological

## Abstract

**Background:**

The value of the CT features and quantitative analysis of lung subsolid nodules (SSNs) in the prediction of the pathological grading of lung adenocarcinoma is discussed.

**Methods:**

Clinical data and CT images of 207 cases (216 lesions) with CT manifestations of an SSNs lung adenocarcinoma confirmed by surgery pathology were retrospectively analysed. The pathological results were divided into three groups, including atypical adenomatous hyperplasia (AAH)/adenocarcinoma in situ (AIS), minimally invasive adenocarcinoma (MIA) and invasive adenocarcinoma (IAC). Then, the quantitative and qualitative data of these nodules were compared and analysed.

**Results:**

The mean size, maximum diameter, mean CT value and maximum CT value of the nodules were significantly different among the three groups of AAH/AIS, MIA and IAC and were different between the paired groups (AAH/AIS and MIA or MIA and IAC) (*P* < 0.05). The critical values of the above indicators between AAH/AIS and MIA were 10.05 mm, 11.16 mm, − 548.00 HU and − 419.74 HU. The critical values of the above indicators between MIA and IAC were 14.42 mm, 16.48 mm, − 364.59 HU and − 16.98 HU. The binary logistic regression analysis of the features with the statistical significance showed that the regression model between AAH/AIS and MIA is *logit(p) = − 0.93 + 0.216X*^*1*^ *+ 0.004X*^*4*^. The regression model between MIA and IAC is *logit(p) = − 1.242–1.428X*^*5*^*(1) − 1.458X*^*6*^*(1) + 1.146X*^*7*^*(1) + 0.272X*^*2*^ *+ 0.005X*^*3*^*.* The areas under the curve (AUC) obtained by plotting the receiver operating characteristic curve (ROC) using the regression probabilities of regression models I and II were 0.815 and 0.931.

**Conclusions:**

Preoperative prediction of pathological classification of CT image features has important guiding value for clinical management. Correct diagnosis results can effectively improve the patient survival rate. Through comprehensive analysis of the CT features and qualitative data of SSNs, the diagnostic accuracy of SSNs can be effectively improved. The logistic regression model established in this study can better predict the pathological classification of SSNs lung adenocarcinoma on CT, and the predictive value is significantly higher than the independent use of each quantitative factor.

## Background

Lung cancer is one of the most common cancers in both male and female, as well as a leading cause of cancer-related death worldwide. Lung adenocarcinoma is the most common subtype of lung cancer [1]. With the gradual promotion and popularization of low-dose CT lung cancer screening of the chest, the detection rate of pulmonary nodules has gradually increased, especially sub-solid nodules (SSNs), also called ground glass nodules (GGNs). Based on the presence of solid components in the nodules, SSNs are divided into two types: mixed ground glass nodules (mGGNs) and pure ground glass nodules (pGGNs) [2]. When a GGN persists for 3 months, it is considered to be associated with lung adenocarcinoma [3]. According to the WHO lung adenocarcinoma pathological classification [4], lung adenocarcinoma is divided into pre-invasive lesions [atypical adenomatous hyperplasia (AAH), (adenocarcinoma in situ (AIS)], minimally invasive adenocarcinoma (MIA) and invasive adenocarcinoma (IAC). Preoperative prediction of pathological classification of CT image features has important guiding value for clinical management. Correct diagnosis results can effectively improve the patient survival rate. Thus the purpose of this study was to evaluate whether the CT features and qualitative data model can predict the pathological classification of SSNs lung adenocarcinoma.

## Patients and methods

### Patients

A retrospective analysis was conducted of 207 cases (216 nodules) of lung adenocarcinoma patients that were surgically and pathologically confirmed and CT findings of SSNs from August 2014 to June 2018 in our hospital. The institutional review board of the First Affiliated Hosptial of Anhui Medical University approved this retrospective study and waived the requirement for informed consent. There were 80 men and 127 women with an age ranging from 33 to 82 years old, with an average age of 57.07 ± 9.39 years old. There were 68 pre-invasive lesions (AIS and AHH), 55 MIA lesions, and 93 IAC lesions. Among them, there were 136 purely ground glass nodules and 80 mixed ground glass nodules. Inclusion criteria: (1) CT examination within 1 month before surgery; (2) maximum diameter of the selected lesions was ≤3 cm; (3) complete preoperative CT images in PACS memory, thin layer reconstruction layer thickness ≤ 1 mm; and (4) no biopsy or any anti-tumour treatment was performed before CT examination.

### CT scan protocol

Chest CT imaging (field-of-view from the apex to the lung basis, including the chest wall and axilla) was performed on Toshiba Aquilion 16-slice CT and 64 detector CT system (GE Discovery CT750 HD or GE Light speed VCT, GE Healthcare)。All parameters were obtained from CT ordinary scan images and reconstructed using standard algorithms. Scanning parameters: GE LightSpeed VCT and GE Discovery CT750 CT equipment were applied with a tube voltage of 120 kV, tube current automatic regulation, layer thickness of 5.0 mm, reconstruction layer thickness of 0.625 mm, pitch 1.375; and the Toshiba Aquilion 16-slice CT equipment application was a tube voltage of 120 kV, tube current of 150 mA, layer thickness of 5.0 mm, reconstruction layer thickness of 1.0 mm, and pitch 0.980.

### Image analysis

Imaging analysis was conducted by two experienced thoracic radiologists (with more than 15 years of imaging diagnosis experience) who were blinded to the pathological results and the patient’s clinical data. The lung window (window width is 1500 HU, window level is − 500 HU) and the mediastinum window (window width is 400 HU, window level is 40 HU) were selected for observation. The window width and window level were fine-tuned for best observing part of the CT features and best observing the bronchogram or the vasculature through multi-planar reconstruction (MPR), maximum density projection (MIP) and minimum density projection (MinIP).

1. Quantitative data (1) mean size (mm): the average of the three sizes (the maximum diameter of the largest slice of the axial image of the lesion and the vertical diameter, the maximum diameter of the lesion in the coronal image) was defined as the mean size. (2) maximum diameter (mm): the maximum diameter of the lesion displayed by multi-directional observation on the MPR lung window image. (3) mean CT value (HU): after selecting the standard axial, sagittal and coronal lung window images and after using the irregular graph tool to draw the boundary of the nodule to be the region of interest (ROI), we recorded the CT value. For the mixed ground glass nodules, the selected measurement image was as large as possible for the largest image of the nodule and the maximum image of the solid part within it. The CT value of each area was recorded, and the average of the three values represents the mean CT value of the lesion. (4) maximum CT value (HU): when the ROI was 10 mm^2^, repeated measurement of areas with high lesion density was performed and the maximum value was taken. 2. Qualitative parameters (Table 1)

**Table 1:** Qualitative parameters CT features of the SSNs

Qualitative parameters	Characteristics
Shape	round/oval, irregular
Edge	lobulation, spiculation, spine-like process
Internal	air-containing space, air bronchogram
Vascular	vascular crossing, vascular change (thickened, twist, convergence)
Pleural indentation	+/−
Tumour-lung interface	clear, blurry

### Pathological diagnosis

The pathological diagnosis and categorization of AAH, AIS, MIA, and IAC were made based on the new pulmonary adenocarcinoma classification,2011 edition [5]. The pathological diagnoses were based on the surgical specimen. Two senior pathologists (with more than 15 years of pathological diagnosis experience) performed all histological preparations and analyses. Any disagreement was discussed and resolved by a mutual consensus or after consultation with a third pathologist.

### Statistical methods

All data were processed by SPSS 17.0 software (IBM Corp, NY, USA). All quantitative data were tested to see if they satisfied the normal distribution and homogeneity of variance and were expressed as $\overline{x}$ ± s. Quantitative data were analysed by one-way ANOVA for comparison among the three groups and Bonferroni tests for comparison between paired groups. The Pearson *X*^2^ test and Fisher’s test (expected value < 5) were used for the qualitative data and multiple comparisons between groups. Quantitative data and qualitative data with statistically significant differences between groups (*P* < 0.05) were used to perform binary logistic regression analysis (advance method) and preserved predictive probability values, and then the predicted probability values, mean size, longest path, mean CT value, and the maximum CT value were used to plot the receiver operating characteristic (ROC) curve. The area under the curve (AUC) was calculated, and we obtained the critical value, sensitivity and specificity of each variable.

## Results

### Basic data and quantitative data statistics and analysis of SSNs (Table 2)

Among the 207 patients, there were 216 SSNs. The mean size, maximum diameter, mean CT value, and maximum CT value of the SSNs were statistically significantly different among AAH/AIS, MIA, IAC and between each paired comparison (*p* < 0.001).

**Table 2:** SSNs quantitative data statistics and analysis results

Variable	AAH/AIS (*n* = 68)	MIA (*n* = 55)	IAC (*n* = 93)	*P* Value
Mean size (mm)	9.19 ± 3.14	12.42 ± 4.19	18.00 ± 4.70	< 0.001
Maximum diameter (mm)	10.49 ± 3.90	14.21 ± 4.97	21.60 ± 5.15	< 0.001
Mean CT value (HU)	− 590.27 ± 127.93	− 473.22 ± 147.01	− 333.32 ± 150.76	< 0.001
Maximum CT value (HU)	− 498.05 ± 201.73	− 288.41 ± 243.37	− 104.825 ± 209.75	< 0.001

*P* < 0.05 was considered significant. The quantitative data were statistically significant between each paired comparison

### The qualitative data analysis of SSNs (Table 3)

**Fig. 1:**
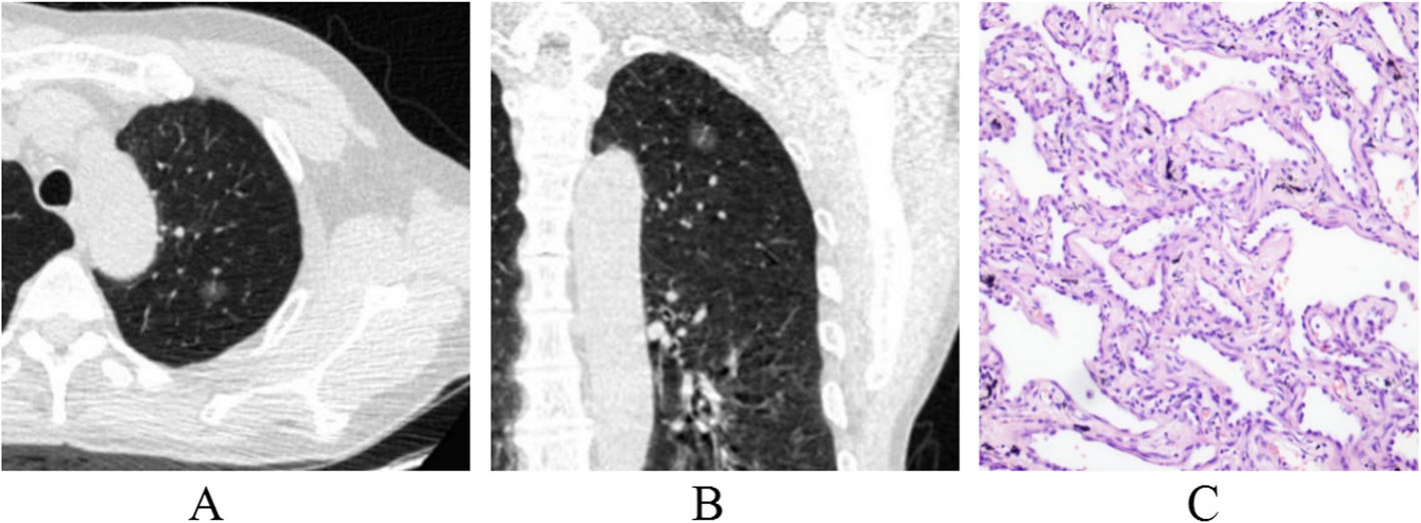
Atypical adenomatous hyperplasia (AAH). One ground-glass nodule was found in the upper lobe of the left lung; its shape is similar to a circle, and the boundary is clear (**a, b**). Pathology (**c**): alveolar epithelial cells changing into atypical adenomatous hyperplasia are observed under a microscope. (HE staining, × 200)

**Fig. 2:**
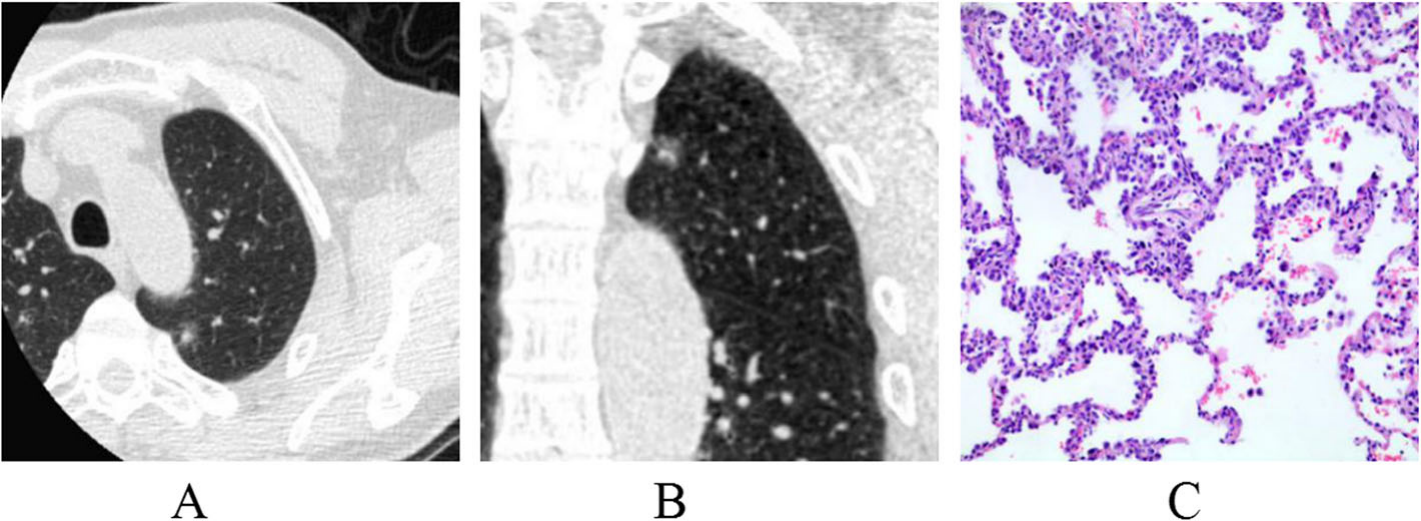
Adenocarcinoma in situ (AIS). One mixed ground-glass nodule was found in the upper lobe of the left lung; its shape is irregular, part of the boundary is blurry, and the adjacent pleura is deformed by traction (**a, b**). Pathology (**c**): the tumour cells adhered to the alveolar cell wall, and the basement membrane was intact as observed under a microscope. (HE staining, × 200)

**Fig. 3:**
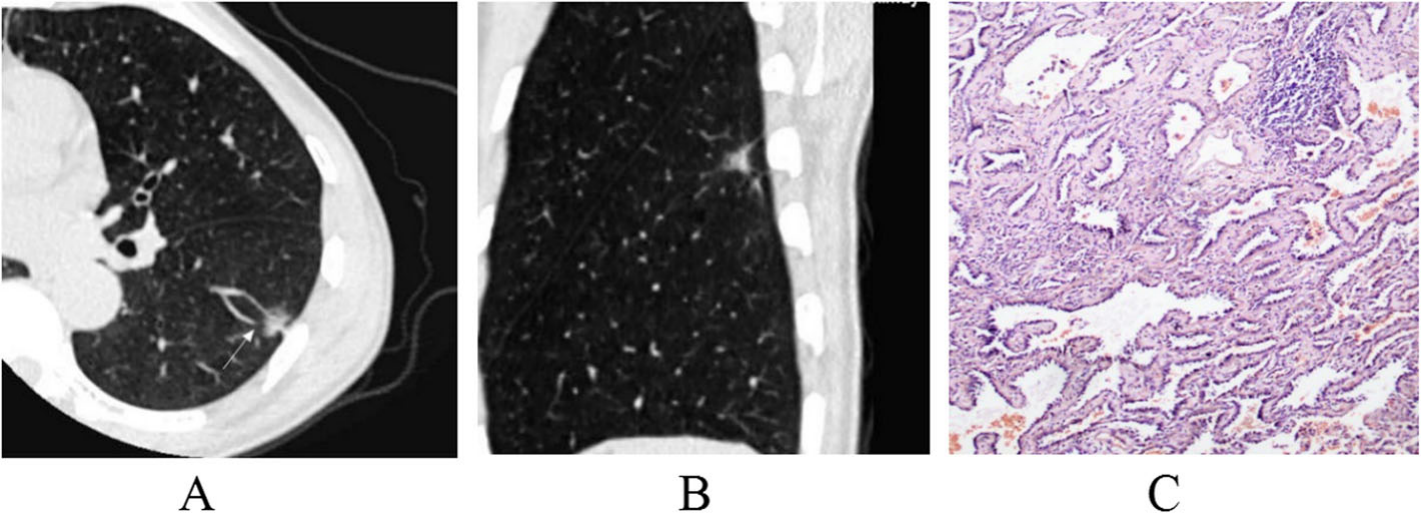
Minimally invasive adenocarcinoma (MIA). One mixed ground glass nodule was found in the lower lobe of the left lung; its shape is irregular, part of the boundary is blurry, and there is vessel convergence around the nodule (**a, b**). Pathology (**c**): tumour cells have infiltrated in the interstitium of the alveolar cells, and the infiltration diameter is < 5 mm. (HE staining, × 200)

**Fig. 4:**
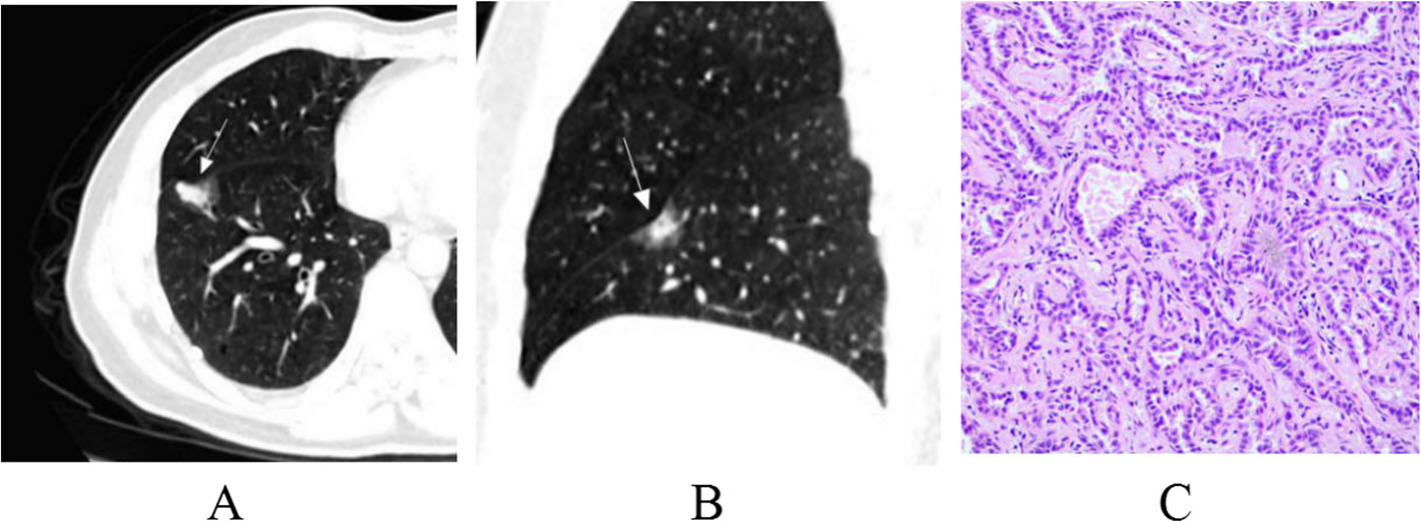
Invasive adenocarcinoma (IAC). One mixed ground glass nodule was found in the lower lobe of the right lung; its shape is irregular, part of the boundary is blurry, and the adjacent pleura is deformed (**a, b**). Pathology (**c**): the alveolar cells are destroyed, and a large number of tumour cells have infiltrated the interstitium. (HE staining, × 200)

The lesion shape, lobulation, spiculation, spine-like process, air-containing space, air bronchogram, vascular changes, pleural indentation and interface among AAH/AIS, MIA and IAC were statistically significant (*p* < 0.001). From AAH/AIS (Figs. 1a-c, 2a-c), to MIA (Fig. 3a-c), to IAC (Fig. 4a-c), with the degree of infiltration increasing, the lesions changed from round or oval and became progressively irregular, and lobulation, spiculation, spine-like processes, air-containing space, air bronchogram, vascular changes, and pleural indentation probability increased gradually, and the tumour-lung interface gradually became clear, but the vascular crossings were not significantly different (*p* > 0.05). There were significant differences between AAH/AIS and MIA for spiculation, air-containing space, air bronchogram, and pleural indentation (*p* < 0.001). Shape, lobulation, spiculation, air bronchogram, vascular changes, pleural indentation and interface characteristics were significantly different (*p* < 0.001) between MIA and IAC.

**Table 3:** SSNs qualitative data statistics and analysis results

Characteristics	AAH/AIS(*n* = 68)	MIA (*n* = 55)	IAC (*n* = 93)	*P* Value
Shape (n)				< 0.001
round/oval	40	34	25^b^	
irregular	28	21	68^b^	
Edge				
lobulation	5	4	50^b^	< 0.001
spiculation	12	22^a^	74^b^	< 0.001
spine-like process	1	1	13	0.002
Internal				
air-containing space	6	16^a^	37	< 0.001
air bronchogram	9	19^a^	58^b^	< 0.001
Vascular				
vascular crossing	56	46	84	0.077
vascular change	27	30	82^b^	< 0.001
Pleural indentation	12	20^a^	72^b^	< 0.001
Tumour-lung interface				< 0.001
clear	27	22	69^b^	
blurry	41	33	24^b^	

^a^Indicates a statistically significant difference between AAH/AIS and MIA

^b^Indicates a statistically significant difference between MIA and IAC

**Fig. 5:**
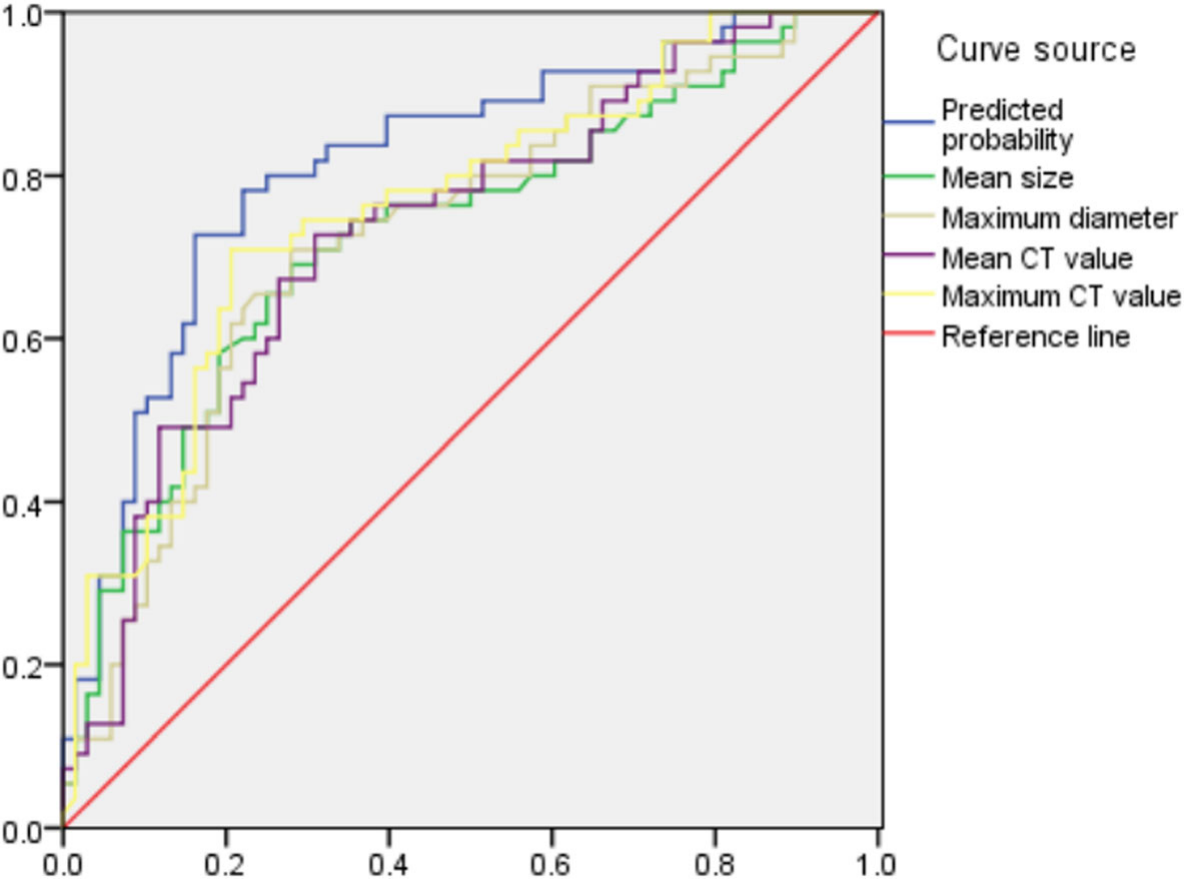
Graph shows ROC curves predicted AAH/AIS and MIA value, Predicted probability, mean size, maximum diameter, mean CT value and maximum CT value, and the areas under the curve for the identification of MIA were 0.815, 0.729, 0.728, 0.733, and 0.761, respectively The area under the curve, sensitivity and specificity were higher than for the single factor analysis indicators of various quantitative parameters

Statistically significant quantitative and qualitative data between the AAH/AIS and MIA groups were analysed by binary logistic regression, and the predicted probability values were saved. The results showed that the mean size (X^1^, *p* < 0.001) and the maximum CT value (X^4^, *p* < 0.001) were the risk factors for judging the AAH/AIS and MIA grouping. Regression model I was logit(p) = −0.93 + 0.216 X^1^ + 0.004X^4^, and the above model was tested by a likelihood ratio test (*p* < 0.001), indicating that the model was statistically significant. Then, we plotted the ROC curves with the predicted probability value, mean size, maximum diameter, mean CT value and maximum CT value (Fig. 5), and the areas under the curve for the identification of MIA were 0.815, 0.729, 0.728, 0.733 and 0.761, respectively (Table 4). The area under the curve, sensitivity and specificity were higher than for the single factor analysis indicators of various quantitative parameters.

**Table 4:** ROC analysis results between groups

Variable	AUC	Sensitivity	Specificity	Threshold
AAH/AIS and MIA				
Mean size	0.729	0.691	0.621	10.05
Maximum diameter	0.728	0.709	0.606	11.16
Mean CT value	0.733	0.727	0.601	−548.00
Maximum CT value	0.761	0.709	0.704	−419.74
Predicted probability	0.815	0.727	0.838	0.478
MIA and IAC				
Mean size	0.809	0.806	0.719	14.42
Maximum diameter	0.874	0.828	0.717	16.48
Mean CT value	0.760	0.656	0.718	−364.59
Maximum CT value	0.739	0.591	0.855	−16.98
Predicted probability	0.931	0.892	0.873	0.580

**Fig. 6:**
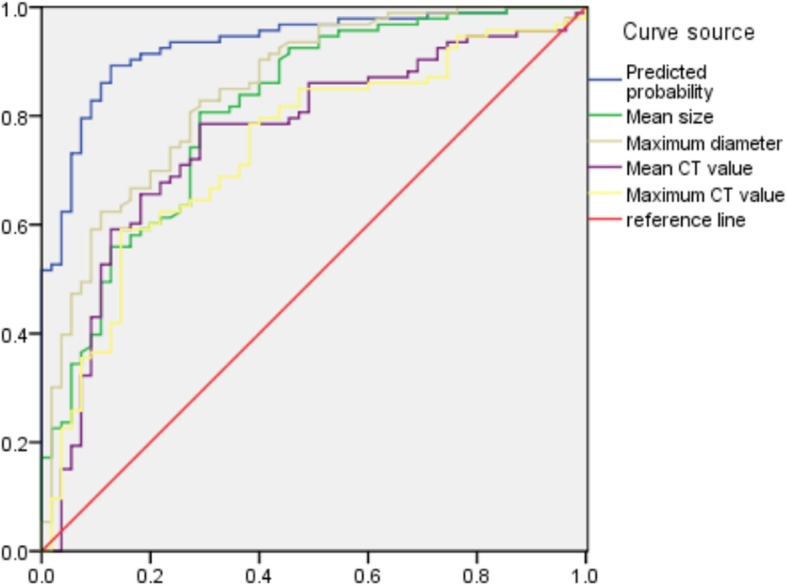
Graph shows ROC curves predicted MIA and IAC value, Predicted probability, mean size, maximum diameter, mean CT value and maximum CT value, and the areas under the curve for the identification of MIA were were 0.931, 0.809, 0.874, 0.760, and 0.739, respectively The area under the curve, sensitivity and specificity were higher than for the single factor analysis indicators of various quantitative parameters

Binary logistic regression analysis was performed on the statistically significant indicators between the MIA and IAC groups, and the predicted probability values were saved. The results showed that lobulated (X^5^, *p* = 0.038), pleural indentation (X^6^, *p* = 0.005), blurred (X^7^, *p* = 0.034), maximum diameter (X^2^, *p* < 0.001), and average CT value (X^3^, *p* = 0.004) were the risk factors for identifying the MIA and IAC grouping. Regression model II was logit(p) = − 1.242–1.428X^5^(1) − 1.458X^6^(1) + 1.146X^7^(1) + 0.272X^2^ + 0.005X^3^, and the above model was tested by a likelihood ratio test (*p* = 0.029), indicating that the model was statistically significant. Then, we plotted the ROC curve with the predicted probability value, mean size, maximum diameter, mean CT value and maximum CT value (Fig. 6). The areas under the curve for the identification of MIA were 0.931, 0.809, 0.874, 0.760, and 0.739, respectively (Table 4). The area under the curve, sensitivity and specificity were higher than the single factor analysis indicators of the various quantitative parameters.

## Discussion

Lung adenocarcinoma is the most important histological subtype of lung cancer. Corresponding to the classification of the WHO lung adenocarcinoma in 2015 [4], early diagnosis and clinical intervention are particularly important for the prognosis of patients. Borczuk et al.’s [6] and Godoy et al.’s [7] studies indicate that the degree of malignancy can be judged by observing changes in the size and density of lung adenocarcinoma lesions. The density of the lesion increases with the degree of infiltration, and the size of the lesion increases with the degree of infiltration. However, the CT features of subsolid nodular lung adenocarcinoma are also important reference indicators in judging the degree of infiltration. In this study, the CT features, size and density of the subsolid nodules and the comprehensive analysis of the influencing factors were used to establish a discriminant model to achieve the possibility of preoperative diagnosis of pathological infiltration grade of lung adenocarcinoma.

### The value of SSNs quantitative data in judging the pathological type of lung adenocarcinoma

Not only can the degree of malignancy be observed by changes in the size and density of lung adenocarcinoma lesions [6, 7], but different density ranges and thresholds can distinguish different pathological types [8]. Invasive adenocarcinoma of the lung has a greater diameter and density than micro-invasive carcinoma and minimally invasive adenocarcinoma [9]. Kitami et al. [10] found that pGGN with a diameter < 1 cm or an average CT value of − 600 HU was used as the critical value for distinguishing between pre-invasive and invasive lesions. Previous study [11] suggested that the mean CT value of − 520 HU as the threshold for distinguishing between MIA and pre-invasive lesions.

The size (mean size and maximum diameter) and CT value of SSNs (mean CT value and maximum CT value) in this study were statistically significant in the three groups of pathological classifications or between each paired comparison. The results of this study are basically consistent with previous literature reports, suggesting that as the degree of pathological infiltration of lesions gradually increases, from AAH/AIS to IAC, the size of the SSNs gradually increases and the density gradually increases. AAH/AIS vs MIA and MIA vs IAC have different sizes, density ranges and thresholds. Therefore, the size and density of SSNs can be used as important indicators for judging the pathology of adenocarcinoma.

### The value of SSNs qualitative data analysis in judging the pathological types of lung adenocarcinoma

The CT features of SSNs are also important indicators for determining the degree of invasion of lung adenocarcinoma. The previous literature reports that various CT features of nodules differ in judging the benign or malignant degree. Ichinose et al. [12] showed that pleural depression was a suggestive factor for malignant tendencies in 191 cases of lesions with pGGN on CT; Hu et al. [13] found that the lobulation and spiculation were significantly different in benign and malignant nodules by studying 112 cases of GGN. FAN et al. [14] analysed 82 cases of focal ground glass nodules (fGGOs) confirmed by pathology, and clinically, the lobulation, spiculation, spine-like process, coarse interface, bronchus cut-off, air-containing space, pleural indentation and vascular convergence of malignant fGGOs were significantly higher than for benign fGGOs.

This study is a comprehensive discussion of the CT features of SSNs, where mainly the morphological features (circular/oval, irregular), marginal features (lobulation, spiculation, spine-like process), internal signs (air-containing space, air bronchogram), pleural indentation, vascular features, and tumour-lung interface (clear, blurry) were analysed, as well as the vascular features including vascular crossing and vascular change (vascular thickening, distortion, aggregation). The results show that various CT features have statistically different degrees of discrimination of the degree of SSNs infiltration, except for vascular crossing.

### The value of SSNs quantitative data and qualitative data analysis for judging the pathological types of lung adenocarcinoma

In the past, there were few studies on comprehensive analysis of various parameters, and there was no simple and discriminant model. To comprehensively analyse the parameters that can be provided by CT images of SSNs, this study comprehensively analysed various quantitative and qualitative data of SSNs. According to the new classification of the WHO lung adenocarcinoma in 2105, model I is logit(p) = − 0.93 + 0.216 X^1^ + 0.004X^4^, established in this study, and the area under the curve for the diagnosis of MIA reached 0.931 between the AAH/AIS and MIA groups. Model II was logit(p) = − 1.242–1.428X^5^(1) − 1.458X^6^(1) + 1.146X^7^(1) + 0.272X^2^ + 0.005X^3^, and the area under the curve for the diagnosis of IAC reached 0.931 between the MIA and IAC groups. The values of model I and model II are higher than the independent determination of each single factor.

There are still some limitations of this study: (1) there are certain selective biases due to using only surgical cases; (2) the accuracy of SSNs quantitative parameters may have a certain impact because of the use of different types of scanning machines, scanning parameters and reconstruction layer thicknesses; and (3) the accuracy of measurement of each indicator may have a certain impact due to personal experience.

## Conclusion

In summary, various quantitative data and qualitative parameters have different degrees of statistical difference in discriminating the pathological classification of SSNs lung adenocarcinoma. Through a comprehensive analysis of CT features and measurement data of SSNs, the diagnostic accuracy of SSNs lung adenocarcinoma can be effectively improved. The logistic regression model established in this study can better predict the pathological classification of SSNs lung adenocarcinoma on CT, and the predictive value is significantly higher than the independent use of each piece of quantitative data. Preoperative prediction of pathological classification of CT image features can improve pre-surgical diagnosis and differential diagnosis. Correct diagnosis results can effectively improve the patient survival rate. In the future study, a prospective clinical trial with more SSNs cases is warranted to further evaluate and validate the diagnostic value of findings in this study.

## Data Availability

Data for this study will not be shared. Sharing data is not included in our research institution review board.

## Abbreviations

AAH: Atypical adenomatous hyperplasia
AIS: Adenocarcinoma in situ
GGNs: Ground glass nodules
IAC: Invasive adenocarcinoma
mGGNs: Mixed ground glass nodules
MIA: Minimally invasive adenocarcinoma
MinIP: Minimum density projection
MIP: Maximum density projection
MPR: Multi-planar reconstruction
pGGNs: Pure ground glass nodules
ROI: Region of interest
SSNs: Sub-solid nodules
